# Cost effectiveness of school-located influenza vaccination programs for elementary and secondary school children

**DOI:** 10.1186/s12913-019-4228-5

**Published:** 2019-06-24

**Authors:** Byung-Kwang Yoo, Stanley J. Schaffer, Sharon G. Humiston, Cynthia M. Rand, Nicolas P. N. Goldstein, Christina S. Albertin, Cathleen Concannon, Peter G. Szilagyi

**Affiliations:** 1 Division of Health Policy and Management, Department of Public Health Sciences, University of California Davis School of Medicine, One Shields Ave. Medical Sciences 1C, Davis, CA 95616 USA; 2 Department of Pediatrics, Golisano Children’s Hospital, University of Rochester School of Medicine and Dentistry, 601 Elmwood Ave, Rochester, NY 14642 USA; 3 Department of Pediatrics, Children’s Mercy Hospital, Kansas City, MO 64108 USA; 4 Department of Pediatrics, UCLA Mattel Children’s Hospital, University of California Los Angeles (UCLA), 10833 Le Conte Avenue, Los Angeles, CA 90095 USA

**Keywords:** School-located vaccination program, Influenza vaccination, Adolescents, School-age children, Web-based consent form system, Cost-effectiveness analysis, Incremental cost-effectiveness ratio

## Abstract

**Background:**

Studies have noted variations in the cost-effectiveness of school-located influenza vaccination (SLIV), but little is known about how SLIV’s cost-effectiveness may vary by targeted age group (e.g., elementary or secondary school students), or vaccine consent process (paper-based or web-based). Further, SLIV’s cost-effectiveness may be impacted by its spillover effect on practice-based vaccination; prior studies have not addressed this issue.

**Methods:**

We performed a cost-effectiveness analysis on two SLIV programs in upstate New York in 2015–2016: (a) elementary school SLIV using a stepped wedge design with schools as clusters (24 suburban and 18 urban schools) and (b) secondary school SLIV using a cluster randomized trial (16 suburban and 4 urban schools). The cost-per-additionally-vaccinated child (i.e., incremental cost-effectiveness ratio (ICER)) was estimated by dividing the incremental SLIV intervention cost by the incremental effectiveness (i.e., the additional number of vaccinated students in intervention schools compared to control schools). We performed deterministic analyses, one-way sensitivity analyses, and probabilistic analyses.

**Results:**

The overall effectiveness measure (proportion of children vaccinated) was 5.7 and 5.5 percentage points higher, respectively, in intervention elementary (52.8%) and secondary schools (48.2%) than grade-matched control schools. SLIV programs vaccinated a small proportion of children in intervention elementary (5.2%) and secondary schools (2.5%). In elementary and secondary schools, the ICER excluding vaccine purchase was $85.71 and $86.51 per-additionally-vaccinated-child, respectively. When additionally accounting for observed spillover impact on practice-based vaccination, the ICER decreased to $80.53 in elementary schools -- decreasing substantially in secondary schools. (to $53.40). These estimates were higher than the published practice-based vaccination cost (median = $25.50, mean = $45.48). Also, these estimates were higher than our 2009–2011 urban SLIV program mean costs ($65) due to additional costs for use of a new web-based consent system ($12.97 per-additionally-vaccinated-child) and higher project coordination costs in 2015–2016. One-way sensitivity analyses showed that ICER estimates were most sensitive to the SLIV effectiveness.

**Conclusions:**

SLIV raises vaccination rates and may increase practice-based vaccination in primary care practices. While these SLIV programs are effective, to be as cost-effective as practice-based vaccination our SLIV programs would need to vaccinate more students and/or lower the costs for consent systems and project coordination.

**Trial Registration:**

ClinicalTrials.gov NCT02227186 (August 25, 2014), updated NCT03137667 (May 2, 2017).

**Electronic supplementary material:**

The online version of this article (10.1186/s12913-019-4228-5) contains supplementary material, which is available to authorized users.

## Background

Seasonal influenza can cause serious morbidity, mortality, and financial burden [1, 2]. Although the United States Advisory Committee on Immunization Practices (ACIP) has recommended universal annual seasonal influenza vaccination for all children aged 6 months to 18 years since 2008 [3], vaccination rates among school-aged children have remained low with little improvement since the 2013–2014 season [4]. For the 2016–17 influenza season, only 60% of 5–12 year olds and 49% of 13–17 year olds received influenza vaccination [4].

One barrier to influenza vaccination is the need for an additional medical visit for vaccination, creating a burden for children and parents [5]. This burden could theoretically be reduced by providing influenza vaccination during school hours, here-in referred to as school-located influenza vaccination (SLIV) [6]. Yet, despite general support for SLIV by pediatricians [7, 8] and parents [9], less than 5% of all child influenza vaccinations were administered at schools during the 2011–2014 seasons [10].

To increase vaccinations in SLIV programs, further data on the effectiveness, the cost-effectiveness, and the financial sustainability of SLIV programs are needed [11, 12]. In our four recent randomized controlled trials (RCTs) of SLIV during the 2009–2010, 2010–2011, 2014–2015, and 2015–2016 vaccination seasons, we found that SLIV increased overall influenza vaccination rates by 5 to 16 percentage points among elementary school children [13, 14] and 5 percentage points among suburban secondary school children [15]. In our model, SLIV clinics were held after November to allow primary care practices to vaccinate many patients before SLIV clinics were held. This ensures that SLIV is a complement to, rather than a substitute for, practice-based vaccination [15, 16]. In our previous cost-effectiveness analysis we found that SLIV cost approximately $65–$73 [in 2015 US dollars] per vaccinated child during the 2009–2011 seasons [17, 18]. Thus, our past SLIV programs were more expensive (i.e., less cost-effective) than (1) practice-based influenza vaccination (median/mean: approximately $25.50/$45.48 per vaccinated child) [17, 19] and (2) seasonal SLIV programs that used other models (e.g., providing donated vaccine without billing or administering vaccine early in the vaccination season) [20–24].

This paper reports the cost-effectiveness analysis (CEA) of SLIV programs in elementary schools [16] and secondary schools (middle and high schools) [15] conducted during the 2015–2016 vaccination season. This paper is novel for several reasons. To our knowledge, this is the first CEA that includes SLIV performed in *secondary* schools (i.e., we and others have performed CEA for SLIV in elementary schools, but not in middle or high schools).

Additionally, this is the first CEA of an SLIV program using an innovative web-based informed consent (hereafter called “web-consent”) system. Web-consent might theoretically reduce SLIV implementation costs by reducing expenses related to copying and handling paper consent forms. If a large proportion of parents use the web-consent that could lower SLIV costs, and SLIV might be more attractive to communities.

The third reason this paper is novel is that we evaluated how the cost-effectiveness of a specific SLIV model can vary based on the potential impact of SLIV upon practice-based vaccination (herein called “spillover”). Spillover can be either *positive spillover* (increasing practice-based vaccination) or *negative spillover* (substituting practice-based vaccination with SLIV) [14–18]. Negative spillover could be a serious barrier to collaborative vaccination delivery between SLIVs and practice-based vaccination [8]. For instance, a national survey among pediatricians reported that 85% of respondents agreed it would be difficult to estimate how much vaccine to order if large numbers of children received influenza vaccine at SLIVs [8]. This is important because practices have to bear financial loss if they are left with unused and non-returnable vaccines [8, 25].

Positive or negative spillover may reflect which particular model of SLIV is used. Specifically, our model started SLIV clinics after November to allow practices to vaccinate as many children as possible first—this would favor positive spillover. Other SLIV programs that started SLIV clinics in early October might result in more of negative spillover or substitution. This is an important point that has not been addressed in prior papers. A key advance in our study is that our SLIV model, while perhaps vaccinating fewer children in school, leads to positive spillover rather than substitution [8, 14–16, 25].

Positive spillover may be related to communication with parents about flu vaccination, with the provision of information about the SLIV program serving as a reminder to parents to take their children to their primary care practices during the vaccination season (even after November when SLIV clinics were held). When positive spillover occurs, measuring the SLIV’s effectiveness by the number of students vaccinated only at SLIV clinics would worsen our SLIV’s cost-effectiveness.

The likelihood and the magnitude of negative spillover depend on the timing of implementing an SLIV program. If an SLIV program is implemented earlier in the influenza vaccination season (e.g., September through November), both the likelihood and the magnitude of negative spillover would be larger than those implemented after November -- thus improving the SLIV program’s cost-effectiveness.

When using an SLIV effectiveness measure of the incremental vaccination rate in SLIV schools compared to control schools (including potential spillover), SLIV cost-effectiveness may vary depending on the direction (either positive or negative) and the magnitude of spillover. Although we previously estimated the cost-effectiveness of our 2009–2010 SLIV program accounting for an observed spillover, we made a simplified assumption that vaccine administration in primary care practices due to spillover did not incur any cost [17]. Instead, the current study assigned the vaccine administration costs related to spillover based on the administration cost at primary care practices [19] (the site at which more than 95% of non-SLIV vaccinations occurred) [16].

Hypotheses:

1. Elementary school SLIV will be more cost-effective than secondary school SLIV. This is based upon the SLIV literature suggesting greater challenges in implementing SLIV (i.e., lower effectiveness) for older children [12]. These challenges to SLIV effectiveness include, for example, less timely receipt of school communication by older students’ parents and less parental perception of the older students’ vulnerability to serious infection.
2. SLIV will create positive spillover. We hypothesize that because parents of students in SLIV schools are exposed to the SLIV-related cues to action (e.g., fliers), they are likely to have their child vaccinated either at school or primary care practices, creating positive spillover.
3. This positive spillover will *substantially* reduce the number of children potentially vaccinated at school. Consequently, the cost-effectiveness of our SLIV program will be (a) worsened if the effectiveness measure *excludes* children vaccinated at primary care practices (i.e., lower effectiveness) and (b) improved if the effectiveness *includes* children vaccinated at primary care practices (i.e., higher effectiveness). Therefore, two effectiveness measures were adopted in our analysis, detailed in the Methods.
4. Elementary and secondary school SLIV will have higher costs and thus be less cost-effective than practice-based influenza vaccination, without accounting for spillover upon practice-based vaccination or without accounting for indirect costs (i.e., parents’ time costs) that reflect a broader societal perspective. However, accounting for these factors, the cost-effectiveness of SLIV may begin to approach that of practice-based vaccination. To test this assumption, our study defined “the standardized program cost” as all costs needed to provide flu vaccination (e.g., reminders), except the cost to purchase vaccine doses. Therefore, for testing hypothesis 4, we applied the common definition of “the standardized program cost” to calculate practice-based vaccination costs from our previously published studies [17, 19]. Hypothesis 4 was developed because the standardized program cost of our SLIV programs explicitly included costs of reminders and informed consent (including the development of the web-consent system) although that of primary care practices did not explicitly include these items [17, 19].
5. This SLIV program that uses a novel web-consent system (including the option to print and turn in a paper consent form) will be more cost-effective than SLIV with a paper-only consent system. We expected that the 2015–2016 web-consent system would reduce the printing, distribution, and handling costs for paper-based consent forms that were observed in our 2009–2011 SLIV programs [18] and would contribute to lower costs of SLIV.

## Methods

### Study design of primary data collection

We collected primary data from two SLIV programs located in elementary schools [16] and secondary schools [15] in Monroe County, New York. Regarding the data from elementary schools, we used a stepped wedge study design with schools as clusters. In 2014–2015, elementary schools were randomly allocated to SLIV or control (control *n* = 10,185 students in 12 suburban and 9 urban schools in Table 1) [16]. Then in 2015–2016, all of the elementary schools were assigned to SLIV (intervention *n* = 21,696 in 24 suburban and 18 urban schools). In secondary schools, we implemented a cluster-randomized trial in 2015–2016 [15]. After selecting 10 pairs of secondary schools (with identical grade levels within pairs), we randomly allocated schools within pairs either to SLIV (intervention *n* = 9488; in 8 suburban and 2 urban schools) or control (control *n* = 8850; in 8 suburban and 2 urban schools) [15].

**Table 1:** Vaccination rates in school-located influenza vaccination program and control schools in 2015–2016

	All Schools^c^				Elementary Schools^c^				Secondary Schools			
	SLIV schools ^b^		Control schools^c^		SLIV schools ^b^		Control schools^c^		SLIV schools ^b^		Control schools	
	Students	% Total Students	Students	% Total Students	Students	% Total Students	Students	% Total Students	Students	% Total Students	Students	% Total Students
TOTAL STUDENTS	31,184	100%	19,035	100%	21,696	100%	10,185	100%	9488	100%	8850	100%
Not vaccinated	15,153	48.6%	10,457	54.9%	10,238	47.2%	5385	52.9%	4915	51.8%	5072	57.3%
Total vaccinated	16,031	51.4%	8578	45.1%	11,458	52.8%	4800	47.1%	4573	48.2%	3778	42.7%
Vaccinated in “practices”^a^	14,665	47.0%	8578	45.1%	10,331	47.6%	4800	47.1%	4334	45.7%	3778	42.7%
Vaccinated at SLIV ^b^	1366	4.4%	n/a	n/a	1127	5.2%	n/a	n/a	239	2.5%	n/a	n/a

^a^ “Practice” indicates the vaccinations’ administration setting other than SLIV clinics, almost always (> 95%) primary care practices [16]

^b^ SLIV: School-located influenza vaccination

^c^ Because of our stepped wedge trial study design, control schools for elementary schools in 2015–2016 were the control schools in 2014–2015

For these two SLIV programs, we notified parents using emails (suburban schools only) or backpack fliers (at both urban and suburban schools). Paper-based vaccination consent forms were available for all schools; these paper-based consent forms included a link to the web-consent system. Web-based consent in our SLIV program worked as follows. Parents received an email about the SLIV program, and the email contained a link to a website. Backpack fliers also contained a written link to the website. The website itself described the SLIV program and included a fillable consent form, which mirrored the Monroe County Department of Public Health (MCDPH) influenza vaccination consent form. Parents completed this form and indicated “yes” to parent consent for influenza vaccination. The information from the website was transmitted to personnel at the MCDPH who checked the New York Immunization Information System (NYSIIS) just prior to the SLIV clinic to ensure the child had not been vaccinated and also reviewed doctor and insurance information.

We partnered with the MCDPH (hereafter called “vendor”), which delegated nurses to administer SLIV vaccinations on a single date per school. We used the NYSIIS data to determine influenza vaccine receipt. Our study’s endpoints were the dates when we matched children’s names and birthdates obtained from the school directories with NYSIIS records to obtain influenza vaccination data: April 1, 2016 for secondary schools [15] and July 31, 2016 for elementary schools [16].

Our earlier publications reported the SLIV program’s effectiveness only, not cost-effectiveness [15, 16]. For instance, the SLIV program’s overall effectiveness measure (proportion of children vaccinated “Anywhere”) was 5.8 and 5.5 percentage points in elementary and secondary schools, respectively, suggesting a moderate impact of SLIV in both elementary and secondary schools [15, 16]. As another effectiveness measure, SLIV programs vaccinated 4.9 and 2.5% of children enrolled in intervention elementary and secondary schools, respectively [15, 16]. It should be noted that the numbers of students vaccinated in the SLIV programs were slightly higher in this manuscript’s Table 1, since the continuing confirmation process of vaccination status identified additionally vaccinated students.

Since influenza immunization rates vary by poverty status [26], we collected data about the percentage of students eligible for free/reduced cost school lunch (i.e., below 185% of the federal poverty level (FPL) [27]) in 2014 (overall 28 and 90% for suburban and urban schools, respectively). This was used among factors for pairing schools within each school district [14]. Compared to the national average of being below 200% FPL (28% of the total population in 2014) [28], our study population’s socio-economic status was slightly lower in suburban schools, but much lower in urban schools.

The Research Subjects Review Board of the University of Rochester approved this study and the protocols for both SLIV programs.

### Cost-effectiveness analysis (CEA)

We developed simple decision tree models to conduct CEAs with the time horizon of one vaccination season (i.e., within 1 year) from the societal perspective, following the guideline by Drummond et al. [29]. Our cost-effectiveness analysis models derived all of the effectiveness parameters and most of the cost parameters from our primary data described above. We calculated the incremental cost-effectiveness ratio (ICER) through dividing the incremental cost (i.e., the difference in cost to vaccinate in intervention schools minus that in control schools) by the incremental effectiveness (i.e., the additional number of vaccinated students in intervention schools compared to control schools irrespective of where students were vaccinated after standardizing the samples sizes of intervention and control schools). The ICER’s unit is dollars-per-additionally-vaccinated-child. We defined effectiveness and cost measures below, following our earlier studies [17, 18].

**Fig. 1:**
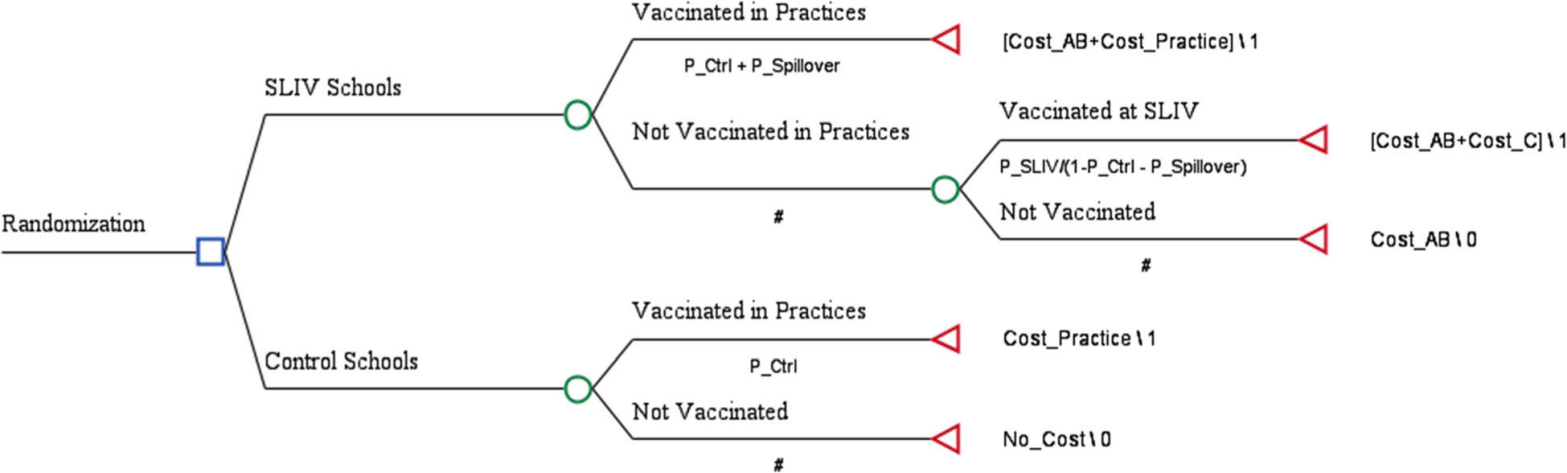
Decision tree model to conduct a cost effectiveness analysis for school-located influenza vaccination program in 2015–2016, accounting for potential spillover. Model parameters are defined in Table 2; # is a probability defined by other parameters so that each chance node (○ in the tree) has the summed probability of 1

**Fig. 2:**
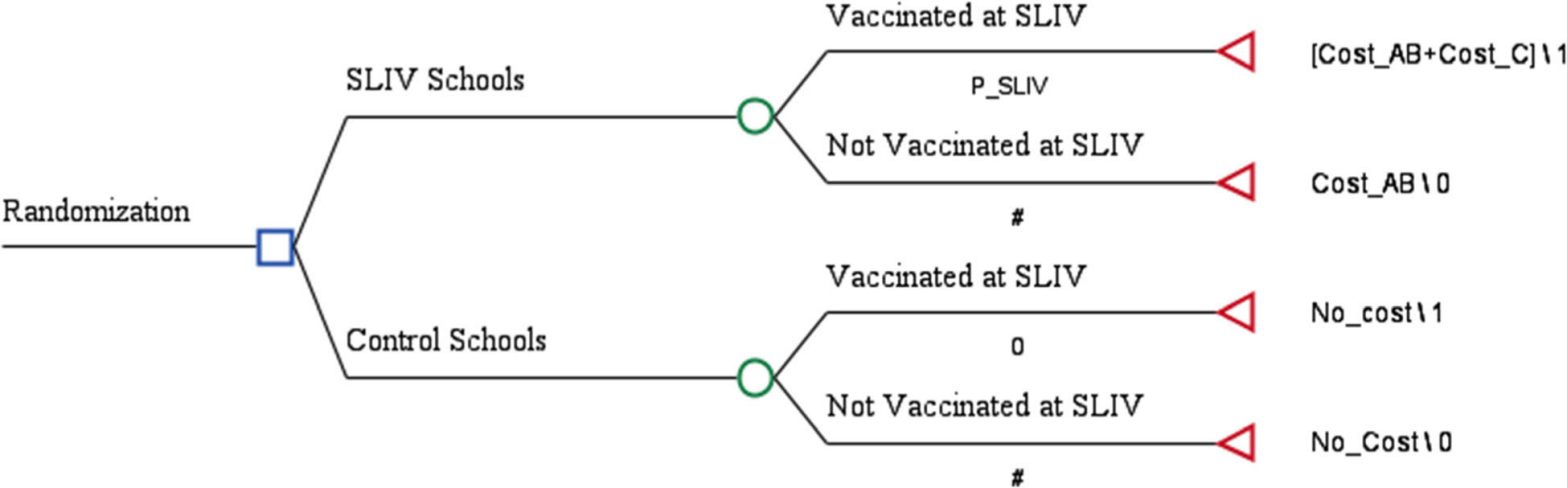
Decision tree model to conduct a cost effectiveness analysis for school-located influenza vaccination program in 2015–2016, without accounting for potential spillover. Model parameters are defined in Table 2; # is a probability defined by other parameters so that each chance node (○ in the tree) has the summed probability of 1

Our CEAs included two sets of deterministic analyses (Figs. 1 and 2). To address the uncertainties of model parameters, we performed a one-way sensitivity analysis and a probabilistic analysis with Monte Carlo simulations by assigning parameters’ distributions (Table 2). For instance, a triangular distribution (mode = 4.4%, minimum = 2.5%, maximum = 5.2%) was assumed for the vaccination rate at SLIV in all schools (Table 2, First row and first column). Monte Carlo simulations allow us to provide the mean and the 95% probabilistic confidence interval (PCI) of the ICER estimates. All simulation analyses used Treeage Pro 2018 (https://www.treeage.com/).

**Table 2:** Parameters for Cost-Effectiveness Analysis Decision Model

Parameter (Name in Decision Trees in Figs. 1 and 2)	All Schools^d^	Elementary Schools^e^	Secondary Schools^f^
Effectiveness Parameters			
Vaccination Rate at SLIV clinics only among Children in SLIV Schools (P_SLIV)	4.4% (2.5%, 5.2%)	5.2% (3.2%, 8.1%)	2.5% (2.0%, 2.6%)
Incremental Vaccination Rate in “Practices^” a^ among Children in SLIV Schools, compared to Vaccination Rate among Children in Control Schools (P_Spillover)	1.9% (0.5%, 3.0%)	0.5% (−1.4%^g^, 1.4%)	3.0% (2.9%, 5.0%)
Vaccinated at Practices among Children in Control Schools (P_Ctrl)	45.1%	47.1%	42.7%
Cost Parameters for All Children in SLIV [$ per eligible child] (Cost_AB = A + B defined below)			
(A) School cost [$ per eligible child]	$1.00 ($0.59, $1.17)	$1.17 ($1.12, $1.26)	$0.59 ($0.58, $0.63)
(B) Project cost [$ per eligible child]	$1.70 ($0.98, $2.02)	$2.02 ($1.82, $2.22)	$0.98 ($0.88, $1.08)
Cost Parameters for Children Vaccinated at SLIV [$ per vaccinated child] (Cost_C = C for “Subtotal Costs”; and Cost_C = C - D for “Total Costs” defined below)			
(C) Vaccine administration	$24.30 ($21.87, $26.73)		
(D) Averted parents’ costs (i.e., to visit primary care practices for a child’s influenza vaccination)^b^	$36.81 ($33.13, $40.49)		
Cost Parameters of Primary Care Practice [$ per vaccinated child] (Cost_Practice) ^c^	$25.50 ($15.68, $45.48)		

All parameters were derived from our original study unless otherwise defined below

SLIV: School-located influenza vaccination

^a^ “Practice” indicates the vaccinations’ administration setting other than SLIV clinics, almost always (> 95%) primary care practices [16]

^b^ This cost component (D) included the cost of parents’ time [30–32] and transportation [33], which does not comprise any medical expenditure, either paid by parents or incurred at primary care practices

^c^ A range of values was derived from our previously published practice-based vaccination cost (25 percentile = $15.68, median = $25.50, and mean = $45.48 [per-vaccinated-child]; Of note, mean (=$45.48) was comparable to 75 percentile (=$45.38)) [17, 19]

^d^ A range of values was derived from the variation between elementary schools and secondary schools

^e^ A range of values was derived from the variation between urban and rural schools within elementary schools

^f^ A range of values was derived from the variation between urban and rural schools within secondary schools

^d,e,f^ A range of values were used as minimum and maximum values to define a triangular distribution (where a mode value is a point estimate) for a parameter distribution used in a probabilistic analysis with Monte Carlo simulations

^g^ A negative value (− 1.40%) presents negative spillover observed among urban elementary schools

### Effectiveness measure

We defined two effectiveness measures in Table 2. Our primary measure (illustrated in Fig. 1) is the difference between the proportion of students vaccinated “Anywhere” among students enrolled in control vs. intervention schools. “Anywhere” includes both vaccinations administered at the SLIV program and those in “practices.” “Practice” indicates the vaccinations’ administration setting other than SLIV clinics, almost always (> 95%) primary care practices [16]. Our definition of “Anywhere” aligned with the past publications [14–18]. This effectiveness measure was used because the SLIV activities could have motivated parents to have their children vaccinated at either a primary care practice or via the SLIV programs.

In Fig. 1, our primary measure is depicted as control school student vaccination (labeled P_Ctrl) vs. intervention school student vaccination -- either at schools (labeled as P_SLIV/(1- P_Ctrl - P_Spillover) among students not vaccinated in practices, (i.e., P_SLIV among all students) or in primary care “practices” (labeled P_Ctrl + P_Spillover). As defined earlier, “spillover” is the potential impact of SLIV upon practice-based vaccination. These model parameters, P_SLIV and P_Spillover, indicate that the SLIV program increased likelihood of vaccination at SLIV and in “practices” for each student enrolled in all SLIV schools.

Our secondary and narrower measure (explained in Fig. 2) is the difference between the proportion of students vaccinated in SLIV vaccination clinics among students enrolled in intervention schools (P_SLIV in Fig. 2) vs. control schools (i.e., zero probability of vaccination at the SLIV program).

Our primary measure captures a more comprehensive effect of the SLIV program than the second one, addressing a potential spillover effect (P_Spillover in Fig. 1) and, hence, potential concern for coordinating with local primary care practices [8, 25]. To our best knowledge, none of past SLIV studies used this comprehensive measure [20–24, 34, 35]. Our secondary and narrower measure was used to compare with these past SLIV studies.

### Cost measures

We estimated all costs in 2015 US dollars, adjusting for changes in the consumer price index (CPI) when necessary [36]. We estimated three components of “Program Costs” incurred by the SLIV program: (A) school costs, (B) project coordination costs, and (C) vendor costs, as well as one “indirect cost” averted by having the SLIV program: (D) averted parents’ costs (i.e., parents’ costs to accompany a child to a primary care practice visit for the child’s influenza vaccination). We defined “Total Cost” as “Program Costs” (A + B + C) minus averted parent costs (D).

#### Program cost components

Component A (school costs) included non-labor material costs (e.g., supplies and expenses for distributing information to parents), labor costs incurred by school staff (e.g., attending preparatory meetings and escorting students), and the web-consent system cost. We estimated labor costs by multiplying the school staff hours self-reported in real-time surveys by the national median wage of a relevant job category as of May 2015 [37]. The annual cost for the web-consent system (shared by all suburban intervention schools) included the annual maintenance cost ($8000) and the upfront equipment cost (i.e., IT costs, averaging the upfront equipment cost of $68,000 over 7 years, i.e., $9714 per year).

Component B (project coordination costs) comprised the cost involved with coordinating activities, but excluded the costs regarding research and evaluation.

Component C (vendor costs) included the vendor’s vaccination administration cost, including (a) labor costs to administer SLIV vaccinations and to bill insurers and (b) material costs including costs for vaccine purchase, the refrigerator for vaccines, and medical supplies in primary analyses. Following the definition of “the standardized program cost,” this Cost Component C did not include the vendor’s vaccine purchase cost.

Component D estimated the averted cost for a household with a child vaccinated through SLIV compared to a household with a child vaccinated outside SLIV. These cost estimates were assumed to be constant across all households. We followed our previous studies [17, 18] in estimating averted parent costs (D), which included parents’ time cost [30–32] and transportation cost [33]. This Cost Component D, $36.81, does not comprise any medical expenditure, either paid by parents or incurred at primary care practices. Of note, this Cost Component D was incorporated in the evaluation of the costs to the SLIV program that accounts for positive spillover.

### Comparison with practice-based vaccination

Our a priori assumption was that SLIV would be less cost-effective than practice-based vaccination if the estimated ICER for SLIV were above the median/mean ($25.50/$45.48) cost-per-additionally-vaccinated child for practice-based influenza vaccination as reported in our past study [17, 19]. To make this comparison, regardless of the proportion of VFC-eligible children in each vaccination site, we excluded the vaccine purchase cost from both SLIV costs and primary care practices’ costs [17, 19] to be comparable to our “standardized program cost” defined earlier. For another comparison, we subtracted the averted parents’ costs (Component D = $36.81) from the SLIV Program Costs.

## Results

### Cost effectiveness analysis (CEA) (Table 3)

Table 3 shows ICER estimates of SLIV by grade grouping (all, elementary, and secondary) and by vaccination site (SLIV clinic, Anywhere), which enable us to compare the SLIV program’s economic efficiency (a) between schools and (b) with that of practice-based vaccination. Table 3 presents ICERs based on both deterministic and probabilistic analyses. These deterministic and probabilistic analyses did not differ qualitatively in terms of testing our hypotheses. Therefore, the following results section mainly discusses the ICER estimates based on deterministic analyses.

**Table 3:** Estimated Incremental Cost-effectiveness Ratio (ICER) [$ per-additionally-vaccinated-child] of school-located influenza vaccination program in 2015–2016 [2015 dollar value]

Vaccination location	All Schools		Elementary Schools		Secondary Schools	
	SLIV ^b^	Anywhere ^c^	SLIV ^b^	Anywhere ^c^	SLIV ^b^	Anywhere ^c^
ICER using SUBTOTAL COST: (A + B + C)						
Deterministic Analysis	$85.84	$67.17	$85.71	$80.53	$86.51	$53.40
Probabilistic Analysis ^a^ (95% PCI)	$87.95 ($70.26, $112.92)	$68.89 ($53.39, $87.24)	$86.67 ($66.82, $108.54)	$84.18 ($64.20, $118.08)	$91.38 ($84.41, $100.98)	$53.53 ($45.57, $61.71)
ICER using TOTAL COST: (A + B + C)-(D)						
Deterministic Analysis	$49.03	$41.76	$48.90	$46.89	$49.70	$36.57
Probabilistic Analysis ^a^ (95% PCI)	$50.17 ($32.46, $77.40)	$43.55 ($30.14, $61.10)	$47.98 ($29.39, $73.91)	$47.70 ($29.44, $75.90)	$54.42 ($46.72, $63.59)	$38.73 ($31.79, $47.38)

All cost estimates were expressed in 2015 dollar value

Cost components (A, B, C, D) are defined in Table 2

^a^ Probabilistic model used the distributions of model parameters defined in Table 2 to perform Monte Carlo simulations to provide the mean and 95% probabilistic confidence interval (PCI) of estimates

^b^ SLIV: School-located influenza vaccination

^c^ “Anywhere” includes both vaccinations administered at the SLIV program and those in “practices.” “Practice” indicates the vaccinations’ administration setting other than SLIV clinics, almost always (> 95%) primary care practices [16]

#### Comparison between elementary and secondary schools

The ICER estimates were comparable between elementary and secondary schools when spillover was not considered. For example, Table 3’s columns of “SLIV” shows that the ICER estimates based on the ‘Subtotal Cost’ (Components A + B + C) were around $86 per-additionally-vaccinated-child in both elementary and secondary schools. However, when spillover was considered, these ICER estimates (in columns of “Anywhere”) indicated that the SLIV programs in secondary schools were more cost-effective than those in elementary schools, regardless of the consideration of Component D.

#### Comparison with practice-based vaccination

As reported above, the ICER estimates (based on Subtotal Cost (Components A + B + C)) were at least $53.40 and $86.51 [per-additionally-vaccinated-child] with and without accounting for spillover, respectively. These estimates were higher than those previously reported for vaccination in primary care practices (mean = $45.48) [17, 19].

When considering indirect averted parent costs (D), our findings changed dramatically. Namely, the ICER estimates (based on Total Cost: Components A + B + C – D, considering spillover) in secondary and all schools ($36.57–$41.76) were lower than the mean cost of vaccination at primary care practices (Table 3). Therefore, our fourth hypothesis – the cost-effectiveness of SLIV may begin to approach that of practice-based vaccination when accounting for spillover and indirect costs – was supported.

#### Comparison with our past SLIV program without the web-consent system

The ICER estimates (based on Subtotal Cost (Components A + B + C) without accounting for spillover) were $85.71 per vaccinated child and $86.51 in elementary and secondary schools, respectively. All of these estimates were higher than our 2009–2011 SLIV cost estimates ($65.14–$65.34). The differences were mainly explained by two costs in 2015–2016: additional cost for web-consent ($0.57 per eligible child and $12.97 per-additionally-vaccinated-child in all SLIV schools; not presented in tables) and higher project coordination cost ($1.70 per eligible child and $38.81 per-additionally-vaccinated-child in all SLIV schools, which was in part related to additional work for the web-consent system; not presented in tables) for each child vaccinated. The web-consent system did not appear to improve SLIV’s economic efficiency except when accounting for spillover. Specifically, when considering spillover, the ICER estimates (based on Subtotal Cost (Components A + B + C)) were higher in 2009–2010 ($72.56), but lower in 2010–2011($63.35), and varied by grade grouping in 2015–2016: all schools ($67.17), elementary schools ($80.53) and secondary schools ($53.40). Thus, our fifth hypothesis – SLIV becomes more cost-effective with the use of the web-consent system – was partly supported only when spillover was considered.

#### One-way sensitivity analysis

Table 4 presents the results of one-way sensitivity analysis of CEA for the SLIV programs in all schools. The ICER estimates were most sensitive to the effectiveness parameters (particularly the primary effectiveness measure, i.e., vaccination rate at SLIV only among children in SLIV schools) but robust to cost parameters. Still, all ICER estimates using Subtotal Cost in Table 4 were higher than the mean cost of practice-based vaccination ($45.48 per vaccinated child). Conversely, ICER estimates, using Total Cost, can be lower than this mean cost for each of seven parameters in Table 4.

**Table 4:** One-way sensitivity analyses for Incremental Cost-effectiveness Ratio (ICER) estimates [$ per-additionally-vaccinated-child] of school-located influenza vaccination program in 2015–2016 [2015 dollar value]

Parameters (Point Estimate)		Range	ICER using Subtotal Cost ^b^		ICER using Total Cost ^b^	
			SLIV ^c^	Anywhere ^d^	SLIV ^c^	Anywhere ^d^
1	Vaccination Rate at SLIV clinics only among Children in SLIV Schools (4.40%)	2.0%	$188.02	$76.74	$151.21	$65.13
		8.10%	$46.42	$52.22	$9.21	$5.22
2	Incremental Vaccination Rate in “Practices^” a^ among Children in SLIV Schools, compared to Vaccination Rate among Children in Control Schools (1.90%)	−1.4%	NA	$114.19	NA	$60.09
		5.0%	NA	$53.68	NA	$36.49
3	SLIV: School cost (= A) [$ per eligible child] ($1.00)	$0.58	$76.35	$60.61	$39.54	$35.19
		$1.26	$91.87	$71.33	$55.06	$45.91
4	SLIV: Project cost (= B) [$ per eligible child] ($1.70)	$0.98	$69.50	$55.88	$32.69	$30.46
		$2.02	$93.24	$72.28	$56.43	$46.86
5	SLIV: Vaccine administration cost (=C) [$ per vaccinated child] ($24.30)	$21.87	$83.42	$65.49	$46.60	$40.07
		$26.73	$88.28	$68.85	$51.46	$43.43
6	Averted parents’ costs (i.e., to visit primary care practices for a child’s influenza vaccination) (=D) [$ per vaccinated child] ($36.81)	$33.13	NA	NA	$52.71	$44.29
		$40.49	NA	NA	$45.35	$39.21
7	Vaccine Administration Cost of Primary Care Practice [$ per vaccinated child] ($25.50)	$15.68	NA	$64.13	NA	$38.71
		$45.48	NA	$73.35	NA	$47.93

*NA* Not Applicable

^a^ “Practice” indicates the vaccinations’ administration setting other than SLIV clinics, almost always (> 95%) primary care practices [16]

^b^ Cost components (Subtotal cost or Total cost) are defined in Table 2

^c^ SLIV: School-located influenza vaccination

^d^ “Anywhere” includes both vaccinations administered at the SLIV program and those in “practices.” “Practice” indicates the vaccinations’ administration setting other than SLIV clinics, almost always (> 95%) primary care practices [16]

#### Break-even analysis

We performed two sets of break-even analyses for CEA:

1. Based on the Subtotal Cost (A + B + C), we estimated the proportion of students vaccinated at SLIV necessary for the cost in SLIV programs and primary care practices to be equivalent, without including averted parents’ costs to visit a medical practice (D). Assuming no change in the magnitude of spillover, this would be achieved if the proportion of children vaccinated in SLIV increased from the actual levels in all schools (4.4%), elementary (5.2%) and secondary (2.5%) to at least 15.1, 17.5 and 8.8%, respectively.
2. Based on the Total Cost [(Program Costs – parent costs) = (A + B + C – D)], we estimated the level of vaccination at SLIV necessary for SLIV to be cost-saving to society (including parents’ averted costs). Assuming no change in the magnitude of spillover, this would be achieved if the proportion of children vaccinated in SLIV clinics increased to at least the following levels: all schools = 11.6%, elementary schools = 12.5%, secondary schools = 8.0%.

## Discussion

SLIV modestly raised vaccination rates in both elementary schools and secondary schools. While these SLIV programs are effective, to be as cost-effective as practice-based vaccination our SLIV programs would need to vaccinate more students and/or lower the costs for consent systems and project coordination. Further, SLIV caused positive spillover, increasing practice-based vaccination among suburban children; this improved the effectiveness and cost-effectiveness of SLIV and should be addressed by future studies. Nonetheless, SLIV was less cost-effective than practice-based vaccination when considering the cost-per-additionally-vaccinated child, but equally or more cost-effective when additionally considering indirect costs (parental time lost from work). Finally, our ICER estimates based on deterministic analyses were robust to one-way sensitivity analyses and probabilistic analyses.

### Hypothesis 1: SLIV in secondary schools and comparison with elementary school SLIV

This is the first published CEA that we can find of a large-scale RCT of an SLIV program administered in secondary schools. Our SLIV program methods in secondary and elementary schools were similar with respect to the procedures used for SLIV vaccine clinics, the use of a shared web-consent system and shared project coordination staff. The results – both overall effectiveness and cost-effectiveness – were also similar for the two groups. It should be noted that the magnitude of the overall effectiveness in secondary schools (5.5% in Table 2, combining first and second rows) was smaller than what was noted in our past RCTs in elementary schools in 2009–2011 [18] and in the literature [12]. Also, the second effectiveness measure (the proportion of school children vaccinated at an SLIV clinic) was lower in secondary schools (2.5% in Table 2, first row) than in elementary schools (5.2% in Table 2, first row). The latter finding appears consistent with a review article indicating that SLIV programs for adolescents tended to be more challenging than those for elementary school students [12].

### Hypotheses 2 & 3: the effect of spillover

Another point of comparison is that our study considered spillover while others did not. Although Effler et al. [22] reported lower cost components than our SLIV programs, their study did not consider a potential negative spillover (reduction in practice-based vaccination) that may have occurred because their large state-wide program started October 15, much earlier than our program in late November. If included, cost-effectiveness of the SLIV program evaluated by Effler et al could have been closer to ours. Similarly, none of the studies listed above [20–24, 35] accounted for a potential spillover in their cost estimations. Only two studies’ limitation sections noted the absence of the data on a potential spillover, [35] which is very important in designing an SLIV program and coordinating with local primary care practices, in addition to evaluating SLIV cost-effectiveness. As noted previously, our SLIV programs offered clinics at the end of November, after primary care practices had a chance to vaccinate many school-aged patients and use up their vaccine supplies [15, 16]. We chose this timing deliberately to minimize potential negative spillover in which SLIV might substitute for practice-based vaccinations [8, 15, 16]. This likely worsened our SLIV program’s cost-effectiveness because more students probably would have been vaccinated at SLIV clinics scheduled earlier in the influenza vaccination season and fewer would have been vaccinated at primary care practices.

Considering spillover would help discuss the SLIV’s potential impact on disparity in vaccination rates between urban (37.3% in control schools in Additional file 1: Table A1) and suburban schools (48.3% in control schools in Additional file 1: Table A1). SLIV decreased such disparity between urban and suburban schools, since SLIV’s secondary effectiveness measure was greater in urban schools (7.2% in Additional file 1: Table A1, bottom row) than suburban schools (2.9% in Additional file 1: Table A1, bottom row). When accounting for spillover, SLIV’s primary effectiveness measure was still greater in urban schools (7.3% (=44.6–37.3%) in Additional file 1: Table A1, third row) than suburban schools (6.5% (=54.8–48.3%) in Additional file 1: Table A1, third row). Comparing these two sets of effectiveness measures, we found that the reduction in disparity between urban and suburban schools was mainly due to the vaccinations at the SLIV clinics rather than positive spillover (0.1% (=37.4–37.3%) in Additional file 1: Table A1, fourth row, in primary care practices) among urban schools. We did not report ICERs specific for urban and suburban schools which were very similar and hence provided little incremental implications.

### Hypothesis 4: comparison with primary care practices and other SLIV programs

Both our elementary SLIV program and our secondary school SLIV program ($53.40–$86.51 per vaccinated child) tend to be much less cost-effective than practice-based influenza vaccination (median/mean = $25.50/$45.48) [17, 19], even considering spillover. Only if we additionally account for indirect averted parental costs to visit primary care practices does our SLIV program in secondary schools ($36.57) become equally or more cost effective than primary care practices. Our SLIV programs --without considering spillover ($85.71–$86.51) -- appear much less cost-effective than other SLIV models when applying the common “standardized program cost” defined earlier (excluding the vaccine purchase cost) and the secondary effectiveness measure: Schmier et al. ($8.03) [20], Hull et al. ($11.63) [21], Cho et al. ($14.93) [34], Effler et al. ($18.01) [22], Kempe et al. ($28.40) [23], Kwong et al.($23.40–$28.67) [24], and Kansagra et al. ($91.41) [35]. Of note, among these studies, only the studies by Cho et al. [34], Effler et al. [22], Kwong et al. [24], and Kansagra et al. [35] reported the detailed cost components that enable a more complete comparison with our SLIV programs.

However, caution is needed for these comparisons. The study on primary care practices [17, 19] did not explicitly include the potential costs for reminders or other costs paid by practices and insurers; our SLIV programs’ components costs (A) and (B) were assumed to be zero in primary care practices. Such cost under-estimation was true for other studies by Cho et al. [34] and Kwong et al. [24]. The former study reported the coordination cost in another publication ($18.27 per vaccinated child), [23, 38] which was lower than our SLIV program’s component cost (A + B = $61.41/$62.21 per-additionally-vaccinated-child in elementary/secondary schools; not presented in tables), but included some clinics without assigning costs for donated materials and uncompensated volunteer staff. In the latter study [24], their vaccine administration costs ($28.67/$23.40 for IIV/LAIV) were comparable to or higher than our SLIV program’s component cost (C), i.e., vendor’s vaccine administration cost ($24.30 in Table 2).

### Hypothesis 5: the effect of web-consent system

Compared to our 2009–2011 SLIV program with paper-only consent, our 2015–2016 SLIV programs with web-consent had lower component costs (A + B, involved with consent processes), and improved cost-effectiveness, only when considering spillover. The web-consent system appears to have dissimilar impacts on the differences in SLIV’s cost-effectiveness’s numerator (i.e., *effectiveness*) and denominator (i.e., *cost*) between 2009 and 2011 and 2015–2016 SLIV programs. Regarding SLIV’s *effectiveness* difference between 2009 and 2011 and 2015–2016, the web-consent system seems to have a small contribution to improving SLIV’s *effectiveness*. This was because use of the web-consent system was not randomly assigned and varied substantially across schools: 4% of urban elementary schools, 73% of suburban secondary schools. Such low use in urban schools may imply that these schools were not ready for the web-based consent. In contrast, SLIV’s *cost* difference between the 2009–2011 and 2015–2016 was largely explained by the additional cost for the web-consent system ($0.57 per eligible child and $12.97 per-additionally-vaccinated-child in all SLIV schools; not presented in tables) and its relevant project coordination cost. Although we did not measure the project coordination cost uniquely spent for the web-consent system (a part of the component cost (B): $1.70 per eligible child and $38.81 per-additionally-vaccinated-child in all SLIV schools; not presented in tables), if this cost was excluded, it is likely that our project coordination costs would have been comparable to those of previous studies.

### Limitations

Our study design could limit the generalizability of our estimates. For instance, we used one county. Yet our vaccination rates in elementary schools (38.9–57.9% in Additional file 1: Table A2, third row) and secondary schools (31.1–50.0% in Additional file 1: Table A3, third row) were slightly lower than or comparable to vaccination rates reported nationally (61.8 and 46.8% for elementary and secondary schools, respectively) [4]. It should be noted that our vaccination estimates among elementary schools were county-representative, since we included all students in these school districts [14]. Also, we examined a relatively small number of secondary schools, although our study population was still one of the largest populations in published studies of SLIV. Additionally, we purposefully scheduled SLIV clinics for late in the vaccination season to allow practices to vaccinate many children, which affected the cost-effectiveness of our SLIV programs as described earlier. Finally, we were unable to obtain school absenteeism rates for this study. However, studies funded by industry (that included free vaccines and substantial labor support, and hence not as “real-world” as our study) have evaluated school absenteeism with mixed results—for example King et al. found no impact on school absenteeism [39] whereas Pannaraj et al. did find an impact [40].

## Conclusions

SLIV modestly raised vaccination rates in both elementary schools and secondary schools. SLIV caused positive spillover, increasing practice-based vaccination in the suburbs; this improved the effectiveness and cost-effectiveness of SLIV and should be addressed by future studies. Nevertheless, SLIV was costlier than practice-based vaccination when only considering the cost-per-additionally-vaccinated child and direct costs. SLIV was comparable to (or less costly than) practice-based vaccination when additionally considering indirect costs (lost parental income due to time lost from work). Considering only direct costs, to be as cost-effective as practice-based vaccination, our SLIV programs would need to vaccinate more students and/or lower project coordination and web-consent system costs.

## Additional file

Additional file 1: **Additional Table A1.** Vaccination rates in school-located influenza vaccination program and control schools in 2015–2016. **Additional Table A2.** Vaccination rates in school-located influenza vaccination program in 2015–2016 (elementary schools only). **Additional Table A3.** Vaccination rates in school-located influenza vaccination program in 2015–2016 (secondary schools only). (DOCX 31 kb)

## Data Availability

The datasets used and analyzed during the current study are available, once de-identified, from the corresponding author on reasonable request.

## Abbreviations

ACIP: Advisory Committee on Immunization Practices
CEA: Cost-effectiveness analysis
ICER: Incremental cost-effectiveness ratio
IIV: Inactivated influenza vaccines
LAIV: Live attenuated influenza vaccine
RCT: Randomized controlled trial
SLIV: School-located influenza vaccination
VFC: Vaccines-For-Children
