# Supplementary material for: Cost effectiveness of school-located influenza vaccination programs for elementary and secondary school children
Source: BMC Health Serv Res. 2019 Jun 24;19:407. doi: 10.1186/s12913-019-4228-5 (PMC6591987; doi:10.1186/s12913-019-4228-5)
Supplement: Supplementary file 1 — Additional Table A1. Vaccination rates in school-located influenza vaccination program and control schools in 2015–2016. Additional Table A2. Vaccination rates in school-located influenza vaccination program in 2015–2016 (elementary schools only). Additional Table A3. Vaccination rates in school-located influenza vaccination program in 2015–2016 (secondary schools only). (DOCX 31 kb) [file 12913_2019_4228_MOESM1_ESM.docx]

**Additional Tables**

**Additional Table A1 Vaccination rates in school-located influenza vaccination program and control schools in 2015–2016**

|  | Suburban + Urban  (already presented in Table 1 above) | | | | Suburban | | | | Urban | | | |
| --- | --- | --- | --- | --- | --- | --- | --- | --- | --- | --- | --- | --- |
|  | SLIV schools ^b^ | | Control schools ^c^ | | SLIV schools ^b^ | | Control schools ^c^ | | SLIV schools ^b^ | | Control schools ^c^ | |
|  | Students | % Total Students | Students | % Total Students | Students | % Total Students | Students | % Total Students | Students | % Total Students | Students | % Total Students |
| TOTAL STUDENTS | 31,184 | 100% | 19,035 | 100% | 20,747 | 100% | 13,477 | 100% | 10,437 | 100% | 5,558 | 100% |
| Not vaccinated | 15,153 | 48.6% | 10,457 | 54.9% | 9,369 | 45.2% | 6,970 | 51.7% | 5,784 | 55.4% | 3,487 | 62.7% |
| Total vaccinated | 16,031 | 51.4% | 8,578 | 45.1% | 11,378 | 54.8% | 6,507 | 48.3% | 4,653 | 44.6% | 2,071 | 37.3% |
| Vaccinated in “practices”^a^ | 14,665 | 47.0% | 8,578 | 45.1% | 10,767 | 51.9% | 6,507 | 48.3% | 3,898 | 37.4% | 2,071 | 37.3% |
| Vaccinated at SLIV ^b^ | 1,366 | 4.4% | n/a | n/a | 611 | 2.9% | 0 | 0.0% | 755 | 7.2% | 0 | 0.0% |

^a^ “Practice” indicates the vaccinations’ administration setting other than SLIV clinics, almost always (>95%) primary care practices [16].

^b^ SLIV: School-located influenza vaccination

^c^ Because of our stepped wedge trial study design, control schools for elementary schools in 2015-2016 were the control schools in 2014-2015.

**Additional Table A2 Vaccination rates in school-located influenza vaccination program in 2015–2016 (elementary schools only)**

|  | Suburban + Urban  (already presented in Table 1 above) | | | | Suburban | | | | Urban | | | |
| --- | --- | --- | --- | --- | --- | --- | --- | --- | --- | --- | --- | --- |
|  | SLIV schools | | Control schools ^c^ | | SLIV schools | | Control schools ^c^ | | SLIV schools | | Control schools ^c^ | |
|  | Students | % Total Students | Students | % Total Students | Students | % Total Students | Students | % Total Students | Students | % Total Students | Students | % Total Students |
| TOTAL STUDENTS | 21,696 | 100% | 10,185 | 100% | 12,691 | 100% | 5,781 | 100% | 9,005 | 100% | 4,404 | 100% |
| Not vaccinated | 10,238 | 47.2% | 5,385 | 52.9% | 5,340 | 42.1% | 2,693 | 46.6% | 4,898 | 54.4% | 2,692 | 61.1% |
| Total vaccinated | 11,458 | 52.8% | 4,800 | 47.1% | 7,351 | 57.9% | 3,088 | 53.4% | 4,107 | 45.6% | 1,712 | 38.9% |
| Vaccinated in “practices”^a^ | 10,331 | 47.6% | 4,800 | 47.1% | 6,950 | 54.8% | 3,088 | 53.4% | 3,381 | 37.5% | 1,712 | 38.9% |
| Vaccinated at SLIV ^b^ | 1,127 | 5.2% | n/a | n/a | 401 | 3.2% | 0 | 0.0% | 726 | 8.1% | 0 | 0.0% |

^a^ “Practice” indicates the vaccinations’ administration setting other than SLIV clinics, almost always (>95%) primary care practices [16].

^b^ SLIV: School-located influenza vaccination

^c^ Because of our stepped wedge trial study design, control schools for elementary schools in 2015-2016 were the control schools in 2014-2015.

**Additional Table A3 Vaccination rates in school-located influenza vaccination program in 2015–2016 (secondary schools only)**

|  | Suburban + Urban  (already presented in Table 1 above) | | | | Suburban | | | | Urban | | | |
| --- | --- | --- | --- | --- | --- | --- | --- | --- | --- | --- | --- | --- |
|  | SLIV schools | | Control schools ^c^ | | SLIV schools | | Control schools ^c^ | | SLIV schools | | Control schools ^c^ | |
|  | Students | % Total Students | Students | % Total Students | Students | % Total Students | Students | % Total Students | Students | % Total Students | Students | % Total Students |
| TOTAL STUDENTS | 9,488 | 100% | 8,850 | 100% | 8,056 | 100% | 7,696 | 100% | 1,432 | 100% | 1,154 | 100% |
| Not vaccinated | 4,915 | 51.8% | 5,072 | 57.3% | 4,029 | 50.0% | 4,277 | 55.6% | 886 | 61.9% | 795 | 68.9% |
| Total vaccinated | 4,573 | 48.2% | 3,778 | 42.7% | 4,027 | 50.0% | 3,419 | 44.4% | 546 | 38.1% | 359 | 31.1% |
| Vaccinated in “practices”^a^ | 4,334 | 45.7% | 3,778 | 42.7% | 3,817 | 47.4% | 3,419 | 44.4% | 517 | 36.1% | 359 | 31.1% |
| Vaccinated at SLIV ^b^ | 239 | 2.5% | n/a | n/a | 210 | 2.6% | 0 | 0.0% | 29 | 2.0% | 0 | 0.0% |

^a^ “Practice” indicates the vaccinations’ administration setting other than SLIV clinics, almost always (>95%) primary care practices [16].

^b^ SLIV: School-located influenza vaccination

^c^ Because of our stepped wedge trial study design, control schools for elementary schools in 2015-2016 were the control schools in 2014-2015.
